# Supplementary material for: WordCommentsAnalyzer: A windows software tool for qualitative research
Source: F1000Res. 2018 Sep 4;7:536. Originally published 2018 May 3. [Version 2] doi: 10.12688/f1000research.14819.2 (PMC6137411; doi:10.12688/f1000research.14819.2)
Supplement: Supplementary file 3 [file f1000research-7-17614-s0002.tgz › b2a79496-4ada-47c4-9682-a99ce867836d.zip!/Eight Iranian Health Journals/Journal of Health System Research _37.docx]

© Journal of Health System Research . This work is licensed under CC BY-NC 4.0 (https://creativecommons.org/licenses/by-nc/4.0/).

Volume & Issue: دوره 8، شماره 2؛1391

Title: آيا مصاحبه تلفني مي‌تواند روش مناسبي براي جمع‌آوري اطلاعات در تحقيقات بهداشتي باشد؟

http://hsr.mui.ac.ir/index.php/jhsr/article/view/354

يكي از مهم‌ترين چالش‌ها براي پژوهشگران، تصميم‌گيري در مورد روش جمع‌آوري اطلاعات مي‌باشد. در مورد استفاده از تلفن براي انجام مصاحبه‌هاي تحقيقاتي نظرات مختلفي وجود دارد. تجربه انجام مصاحبه تلفني در زنان بالاي 40 سال شهر اصفهان در زمينه رفتار غربالگري ماموگرافي نشان داد که تلفن مي‌تواند يك روش مفيد براي جمع‌آوري اطلاعات باشد. این در صورتی است که تركيبي از فرايند ارتباط مؤثر و طراحي چارچوب مناسب براي مصاحبه تلفني وجود داشته باشد. مزاياي مصاحبه تلفني براي جمع‌آوري اطلاعات شامل پوشش مناطق جغرافيايي بيشتر، استفاده از منابع انساني و مالي به طور مؤثر، بهبود كيفيت جمع‌آوري اطلاعات، حفظ امنيت مصاحبه‌كننده، سرعت بيشتر جمع‌آوري اطلاعات و احساس راحتي بيشتر مي‌باشد. مهم‌ترين چالش‌هاي انجام مصاحبه تلفني شامل عدم امكان استفاده از علایم بصري جهت جلب اعتماد مصاحبه‌شونده، عدم برقراري و حفظ ارتباط شفاف، ارایه سؤالات غير مرتبط و مخالفت اعضای خانواده با شركت در مصاحبه تلفني مي‌باشد. شواهد نشان داد كه آمادگي براي انجام مصاحبه تلفني ميزان موفقيت را افزايش مي‌دهد.

Title: بررسی تأثیر آموزش بهداشت بارداری بر آگاهی و نگرش دختران در شرف ازدواج در شهرستان بویراحمد

http://hsr.mui.ac.ir/index.php/jhsr/article/view/605

مقدمه: سالانه در جهان نزدیک به 529000 زن به دنبال عوارض بارداری و زایمان جان خود را از دست می‌دهند. آموزش دختران قبل از ازدواج می‌تواند نقش مؤثری در ارتقای بهداشت دوران بارداری ایفا نماید. انتخاب شیوه آموزشی مناسب به سودمندی بیشتر آموزش می‌انجامد. این مطالعه به بررسی اثر آموزش بهداشت بارداری به شیوه بحث گروهی بر آگاهی و نگرش دختران در شرف ازدواج در شهرستان بویر احمد پرداخته است. روش‌ها: این پژوهش نیمه تجربی در شهرستان یاسوج در سال 1389 انجام شد. از بین شرکت کنندگان در برنامه مشاوره قبل از ازدواج، طی یک سال تعداد 577 نفر به روش نمونه‌گیری در دسترس مستمر انتخاب ‌شدند.  اطلاعات پیش آزمون با پرسشنامه محقق ساخته شامل اطلاعات جمعیت شناختی (سن، تحصیلات و شغل)، 10 سؤال آگاهی و 10 سؤال نگرشی در زمینه بهداشت بارداری جمع‌آوری ‌شد. سپس مداخله آموزشی طی دو جلسه، هر هفته یک جلسه یک و نیم ساعته به شیوه بحث گروهی و پرسش و پاسخ توسط دانشجویان بهداشت خانواده اجرا ‌گردید. یک ماه بعد از مداخله دوباره اطلاعات جمع‌آوری ‌شد و با قبل از آموزش مقایسه ‌گردید. یافته‌ها: میانگین سن دختران در شرف ازدواج 5/4 ± 1/22 به دست آمد. 3/84 درصد از دختران خانه‌دار و 7/15 درصد شاغل بودند. میانگین نمرات آگاهی و نگرش دختران دارای تحصیلات دانشگاهی به طور معنی‌داری بیشتر از سایرین بود. بعد از آموزش میانگین نمرات آگاهی و نگرش دختران به طور معنی‌داری افزایش یافته بود (001/0 ≤ P). نتیجه‌گیری: آموزش به شیوه بحث گروهی و پرسش و پاسخ باعث افزایش معنی‌دار سطح آگاهی و نگرش دختران شده بود. با توجه به اهمیت و لزوم آموزش بهداشت بارداری، پیشنهاد می‌گردد جهت استفاده بهتر از این فرصت آموزشی از بحث گروهی و پرسش و پاسخ و جلب مشارکت فعال دختران استفاده گردد.

Title: كاربرد مدل اعتقاد بهداشتي در پيش‌گيري از پوكي استخوان در رابطين بهداشت مراكز بهداشتي- ‌درماني شهر خرم‌آباد

http://hsr.mui.ac.ir/index.php/jhsr/article/view/606

مقدمه: استئوپروز شايع‌ترين بيماري متابوليک استخوان است كه با ايجاد اختلال ساختاري در استخوان، زمينه را براي بروز شكستگي استخوان مستعد مي‌سازد. این مطالعه به منظور بررسی تأثیر مدل اعتقاد بهداشتی در پیش‌گیری از استئوپروز در رابطین بهداشت شهرستان خرم‌آباد در سال 90-1389 انجام گردید. روش‌ها: مطالعه حاضر نیمه تجربی از نوع قبل و بعد بود که بر روی 100 رابط بهداشتی که به صورت تصادفی به دو گروه 50 نفره آزمون و شاهد تقسیم شده بودند، انجام گرفت. سپس در طي سه مرحله (قبل از مداخله، بلافاصله بعد از مداخله و سه ماه بعد ار مداخله) مورد ارزيابي قرار گرفتند. تجزيه و تحليل داده‌ها با استفاده از نرم‌افزار 18SPSS و توسط آزمون Repeated measurement صورت گرفت. یافته‌ها: قبل از مداخله تفاوت معنی‌داری بین میانگین نمرات سازه‌های مختلف این مدل در دو گروه مشاهده نشد. اما بعد از مداخله در گروه آزمون میانگین نمره‌ آگاهی و سازه‌های مختلف مدل (حساسیت درک شده، شدت درک شده، منافع درک شده و موانع درک شده) به طرز معنی‌داری افزایش پیدا کرد (05/0 > P). اما در بخش عملکرد، مصرف کلسیم در هر دو گروه آزمون و شاهد کاهش یافت. نتیجه‌گیری: مدل اعتقاد بهداشتی با آن که برای طرح‌ریزی برنامه‌هایی جهت پیش‌گیری از بیماری و سوانح مفید می‌باشد، لیکن به طور ایده‌آل برای ارتقای رفتارها به ویژه تغییر رفتار طولانی مدت و رفتارهایی که به عوامل اقتصادی و اجتماعی وابسته‌اند مناسب به نظر نمی‌رسد.

Title: بررسی شیوع فشار خون بالا و دیابت و عوامل مرتبط با ابتلا به آن‌ها بر مبنای مطالعه‌ای بزرگ روی جمعیت عمومی اصفهان- کاربردی از مدل رگرسیون لجستیک چند متغیره

http://hsr.mui.ac.ir/index.php/jhsr/article/view/607

مقدمه: در اين مطالعه شيوع کلی، سنی و جنسی فشار خون بالا و ديابت نوع 2 برآورد شد. همچنین ارتباط بعضي از عوامل خطر با ابتلای همزمان به این دو بیماری مورد ارزيابي قرار گرفت. روش‌ها: در یک مطالعه مقطعی، 3000 مرد و زن 15 تا 64 ساله اصفهانی که با استفاده از روش نمونه‌گيري طبقه‌بندي همراه با خوشه‌بندي چند مرحله‌ای در بازه زمانی 1386 تا 1388 انتخاب شدند، مورد بررسي قرار گرفتند. اطلاعات مربوط به عوامل خطر بالقوه مربوط به ابتلا به فشار خون بالا و دیابت و نیز وضعیت ابتلا به این بیماری‌ها برای هر آزمودني با استفاده از یک پرسش‌نامه جامع مورد سنجش و اندازه‌گيري قرار گرفت. از روش رگرسيون لجستيك چند متغيره در نرم‌افزار R ویرایش 2.13.2 برای تحلیل داده‌ها استفاده گردید. يافته‌ها: شيوع کلی ديابت نوع 2 برابر 6/6 درصد و شيوع کلی فشار خون بالا برابر 2/22 درصد برآورد گردید. شیوع فشار خون بالا در زنان از مردان بالاتر بود (4/22 درصد در برابر 06/22 درصد، 05/0 < P). همچنین شیوع دیابت در زنان به مراتب بالاتر از مردان بود (27/8 درصد در برابر 86/4 درصد، 01/0 < P). ارتباط ميان سن، جنس، شاخص توده بدني و سابقه فاميلي ابتلا به ديابت با ابتلا به ديابت معني‌دار شد. در مورد فشار خون بالا، ارتباط سن، جنس و شاخص توده بدني و مصرف سیگار معني‌دار گردید. نتيجه‌گيري: مطالعه حاضر نشان داد که شیوع بیماری‌های دیابت و به ویژه فشار خون بالا در جامعه مورد بررسی، بالا است و در مورد هر دو بیماری شیوع در زنان بالاتر است. بخش عمده‌ای از عوامل مرتبط با ابتلا به این بیماری‌ها، متغیرهای قابل کنترل مربوط به سبک زندگی می‌باشد. بنابراين اقدامات در حوزه آموزش عمومی و پيشگيری می‌بايست در جهت ارتقای شیوه زندگی سالم در سطح جامعه همراه با توجه ویژه به زنان معطوف گردند.

Title: بررسي غلظت کلروفرم در شبكه توزيع آب شهر اصفهان و پهنه‌بندی آن با سیستم اطلاعات جغرافیایی (GIS)

http://hsr.mui.ac.ir/index.php/jhsr/article/view/608

مقدمه: این مطالعه با هدف تعیین غلظت کلر باقی‌مانده کل و کلروفرم در شبكه توزيع آب شهر اصفهان و پهنه‌بندی آن با سیستم اطلاعات جغرافیایی (Geographic information system یا GIS) انجام گرفت. روش‌ها: این مطالعه از نوع توصیفی- مقطعی است. کلر باقی‌مانده کل در محل نمونه‌برداری از شبکه توزیع آب اصفهان توسط دستگاه پرتابل و غلظت کلروفرم بعد از انتقال نمونه‌ها در شرایط استاندارد به آزمایشگاه با استفاده از دستگاه طیف‌سنج جرمی (Gas chromatography–mass spectrometry یا GC-MS) اندازه‌گیری شد. برای انجام پهنه‌بندی نتایج به دست آمده از سنجش پارامترهای مورد آنالیز از GIS استفاده گردید. یافته‌ها: مقادیر اندازه‌گیری شده حداکثر و حداقل به ترتیب برای کلر باقی‌مانده کل ppm 3/3 و غیر قابل تشخیص و برای کلروفرم ppb 28 و ppb 10 است که غلظت‌های به نسبت زیاد کلروفرم بیشتر مربوط به نقاط انتهایی شبکه و حاشیه شهر است. نقاط مرکزی شهر دارای غلظت کمتری از کلروفرم نسبت به سایر نقاط است. دلیل این امر را می‌توان به مصرف بیشتر آب در نقاط مرکزی شهر و راکد ماندن آب در نقاط حاشیه‌ای شهر مربوط دانست. نتیجه‌گیری: در حال حاضر آب خروجی از تصفیه‌خانه آب شرب اصفهان کیفیت مناسبی از لحاظ وجود کلر باقی‌مانده و محصولات ثانوی گندزدایی دارد. مقادیر آن در محدوده مجاز WHO (Word health organization) و USEPA (United states environmental protection agency) می‌باشد ولی ضرورت دارد غلظت احتمالي تري هالومتآن‌های توليدي به صورت مستمر اندازه‌گیری شود و با استفاده از روش‌های مناسب تلفیقی براي كاهش تشكيل این ترکیبات مورد توجه قرار گیرد.

Title: ارزیابی مکان دفن جدید پسماندهای شهرستان اصفهان و حومه بر اساس شاخص اولکنو با استفاده از سامانه اطلاعات جغرافیایی (Geographical information systems)

http://hsr.mui.ac.ir/index.php/jhsr/article/view/609

مقدمه: مکان‌های دفن مواد زاید شهری دارای پتانسیل بالایی در آلوده کردن محیط و اثر بر سلامت انسان به واسطه تجزیه مواد زاید و تولید شیرابه و گازهای دی‌اکسیدکربن و متان هستند. در این تحقیق به ارزیابی مکان دفن مواد زاید بر اساس شاخص اولکنو با توجه به میزان بارندگی سالیانه، جنس خاک و فاصله سطح آب زیرزمینی از کف محل دفن در محیط GIS (Geographical information systems) پرداخته شده است. روش‌ها: این تحقیق بر اساس جمع‌آوری داده‌ها و اطلاعات و مطالعات کتابخانه‌ای و ورود داده‌ها و تجزیه و تحلیل آن‌ها به روش هم‌پوشانی لایه‌ها در محیط GIS انجام شد. یافته‌ها: در سال 1389 میزان بارندگی در کل منطقه دفن کمتر از 150 میلی‌متر در سال بوده است. خاک منطقه از جنس رسی لومی بود و متوسط فاصله از کف محل دفن تا سطح آب زیرزمینی 3 تا 9 متر بود. بر اساس نتایج محاسبات به عمل آمده، مکان دفن جدید زباله در شهر اصفهان در منطقه با نمره 40 واقع شده است، یعنی دارای شرایط مناسبی بوده است و پتانسیل آلودگی آب‌های زیر زمینی آن پایین می‌باشد. نتیجه‌گیری: مکان دفن جدید زباله‌های شهرستان اصفهان و شهرک‌های حومه، از نظر شاخص اولکنو مناسب بود و احتمال آلودگی آب زیرزمینی این منطقه توسط شیرابه‌های تولیدی آن نیز بر اساس این شاخص کم پیش‌بینی شد.

Title: بررسي نحوه توزیع بطری‌های کودک پليمري با نشان"بدون بيسفنل‌آ" در جامعه شهری اصفهان

http://hsr.mui.ac.ir/index.php/jhsr/article/view/610

مقدمه: بیسفنل‌آ، ترکیبی افزودنی است که جهت ساخت پلاستیک‌های پلی‌کربناته استفاده می‌شود. به تازگی استفاده از این ماده در تهیه بطری‌های کودکان در بسیاری از کشورها ممنوع شده است. روش‌ها: این مطالعه مقطعی با هدف ارزیابی توزیع بطری‌های پلی‌کربناته و استاندارد کیفی آن‌ها در شهر اصفهان در سال 1390 صورت گرفت. 35 مغازه فروش لوازم کودک و 5 داروخانه در چهار منطقه مختلف اصفهان مورد بررسی قرار گرفت. یافته‌ها: بطری‌ها بر اساس برند، کشور سازنده و عدم وجود بیسفنول‌آ مورد ارزیابی قرار گرفتند. کیفیت محصولات به طور مستقیم به قوانین کشور تولید کننده آن‌ها بستگی داشت؛ به طوری که در سه دسته ایرانی، آسیایی و اروپایی- آمریکایی تقسیم‌بندی گردیدند.بطری‌های ایرانی و اروپایی به ترتیب بیشترین و کمترین میزان توزیع را در بازار تشکیل می‌دادند. تفاوت در کیفیت استاندارد انواع بطری‌ها به مناطق جغرافیایی مورد بررسی آن‌ها مرتبط بود. بطری‌های با علامت "بدون بیسفنول‌آ" در محصولات اروپایی و تنها در دو منطقه اصفهان یافت شدند. نتیجه‌گیری: با توجه به توزیع بالای بطری‌های ایرانی در بازار داخلی چنین می‌توان نتیجه گرفت که بهبود و تجدید نظر در استانداردهای داخلی می‌تواند در کاهش مواجهه با بیسفنل‌آ در نوزادان ایرانی مؤثر باشد. بر این اساس، ارزیابی خطر دریافت بیسفنل‌آ از طریق بطری کودک در جامعه امری ضروری است.

Title: عوامل محیطی مرتبط با سرطان کولورکتال اسپورادیک، بیمارستان سیدالشهدای شهرستان اصفهان

http://hsr.mui.ac.ir/index.php/jhsr/article/view/611

مقدمه: امروزه سرطان یکی از معضلات مهم و اساسی بهداشت و درمان در سراسر دنیا می‌باشد و در کشور ما نیز اهمیت این بیماری رو به افزایش است. به طوری که این بیماری سومین عامل میرایی و دومین گروه بزرگ از بیماری‌های مزمن غیر قابل انتقال را به خود اختصاص داده است. سرطان کولورکتال شایع‌ترین سرطان دستگاه گوارش در سراسر دنیا است و 10 درصد موارد فوت ناشی از سرطان را شامل می‌شود. عوامل ژنتیکی و محیطی متفاوتی در ایجاد این سرطان نقش دارند که در این پژوهش به بررسی عوامل محیطی مرتبط با سرطان کولورکتال در شهرستان اصفهان پرداخته شد. روش‌ها: در این مطالعه مورد- شاهدی، 125 بیمار که نتیجه کولونوسکوپی آن‌ها مثبت بود از تنها مرکز سرطان کولورکتال استان اصفهان (بیمارستان سیدالشهدا) به عنوان مورد و 135 نفر که نتیجه کولونوسکوپی آن‌ها منفی شده بود از همان مرکز به عنوان شاهد انتخاب شدند. موارد و شاهدها از نظر سن، جنس و محل سکونت با هم همسان گردیدند. با توجه به هدف مطالعه جهت بررسی عوامل سرطان کولورکتال اسپورادیک، بیمارانی که از نظر سابقه فامیلی مثبت بودند از مطالعه خارج شدند. اطلاعات استخراج شده شامل سن، جنس، فعالیت، رژیم غذایی، مصرف داروهای غیراستروئیدی، دخانیات و BMI (Body mass index) بود. داده‌ها با استفاده از مدل رگرسیون لجستیک بعد از ورود به نرم‌افزار SPSS مورد تجزیه و تحلیل قرار گرفت. یافته‌ها: از مجموع بیماران مورد بررسی 9/51 درصد مرد، 1/48 درصد زن و میانگین سنی بیماران 002/11 ± 32/58 بود. در آنالیز چندگانه یک ارتباط معکوس بین فعالیت فیزیکی (7/0-34/0 :CI 95 درصد، 49/0 = OR) و مصرف داروهای غیر استروئیدی (2/0-02/0 :CI 95 درصد، 07/0 = OR) با سرطان کولورکتال پیدا شد. همچنین یک ارتباط مثبت بین مصرف رژیم غذایی پرچرب و سرطان کولورکتال پیدا شد (45/5-96/2 :CI 95 درصد، 86/4 = OR). در این مطالعه ارتباطی بین مصرف سیگار و نمایه توده بدنی با سرطان کولورکتال مشاهده نگردید. نتیجه‌گیری: با توجه به قابل پیش‌گیری بودن سرطان کولورکتال و افزایش بروز جهانی بیماری، مداخله‌های آموزشی در خصوص اهمیت بیماری، غربالگری و تشخیص، نقش رژیم غذایی و فعالیت بدنی در ایجاد بیماری ضروری به نظر می‌رسد.

Title: بررسی رفتارهای پیش‌گیری کننده از اعتیاد و سوء مصرف مواد مخدر مبتنی بر الگوی اعتقاد بهداشتی در دانش‌آموزان دبیرستان‌های پسرانه منطقه بروجن

http://hsr.mui.ac.ir/index.php/jhsr/article/view/612

مقدمه: اعتياد به مواد مخدر به عنوان يكي از مشكلات بهداشتي، درماني و اجتماعي قرن حاضر شناخته شده است. مطالعات اخير نشان داده است كه جوانان در برابر مصرف مواد مخدر مقاومت كمي دارند، ولي درباره خطرات ناشي از مصرف مواد، قابليت درك و پذيرش بالايي دارند. این تحقیق با هدف تعیین رفتارهای پیش‌گیری کننده از اعتیاد به مواد مخدر بر اساس مدل اعتقاد بهداشتی در بین دانش‌آموزان انجام شده است. روش‌ها: این بررسی یک مطالعه توصیفی- تحلیلی بود که در بین 224 نفر از دانش‌آموزان مقطع دبیرستان در چهار دبیرستان پسرانه منطقه بروجن انجام گردید. روش نمونه‌گیری در این مطالعه، چند مرحله‌ای تصادفی بود. در این مطالعه توصیفی از متغیرهای دموگرافیک و متغیرهای مبتنی بر سازه‌های مدل اعتقاد بهداشتی (منافع درک شده، شدت درک شده، حساسیت درک شده، موانع درک شده، خود کارامدی و راهنما برای عمل) در جهت عملکردهای پیش‌گیری کننده از اعتیاد و سوء مصرف مواد مخدر استفاده گردید. داده‌های جمع‌آوری شده توسط نرم‌افزار 18SPSS و آزمون‌های توصیفی و استنباطی تجزیه و تحلیل شد. یافته‌ها: بین نمره کسب شده از سازه‌های مدل اعتقاد بهداشتی و رفتارهای پیش‌گیری کننده از سوء مصرف مواد مخدر در دانش‌آموزان همبستگی معنی‌داری وجود داشت. همچنین بین تحصیلات پدر دانش‌آموزان و نمره آگاهی به دست آمده توسط ایشان از عوامل مرتبط با پیش‌گیری از سوء مصرف مواد اعتیادآور (001/0 = P) و میان نحوه بیان مشکلات ایشان و رفتارهای پیش‌گیری کننده از سوء مصرف مواد مخدر، رابطه معنی‌داری (002/0 = P) وجود داشته است. نتیجه‌گیری:. یافته‌های این تحقیق همانند مطالعات مشابه دیگر نشان داد که آگاهی از عوامل مرتبط با پیش‌گیری از اعتیاد و نمره کسب شده از سازه‌های مدل اعتقاد بهداشتی در دانش‌آموزان می‌تواند به طور معنی‌داری بر عملکردهای پیش‌گیری کننده آن‌ها تأثیر بگذارد.

Title: تأثير الگوي توانمند سازي خانواده محور بر دانش، نگرش و عملکرد بيماران مبتلا به مولتيپل اسکلروزيس

http://hsr.mui.ac.ir/index.php/jhsr/article/view/621

مقدمه: هدف از انجام این پژوهش، مداخلات آموزشی در مراقبت خانواده محور و ارتقای توانايي‌هاي بیمار و اعضای خانواده مي‌باشد تا به موانع موجود در زمينه‌هاي بهداشتي و سلامتي فایق آيند. لذا پژوهش حاضر به منظور بررسی تأثیر الگوی توانمند سازی خانواده محور بر دانش، نگرش و عملکرد بیماران مبتلا به مولتیپل اسکلروزیس انجام شده است. روش‌ها: اين مطالعه با طراحي کارآزمايي باليني و با 70 بیمار مبتلا به مولتيپل اسکلروزيس در دو گروه آزمون و شاهد انجام شد. 8 جلسه 60 دقیقه‌ای برنامه آموزشي بر اساس نيازهاي آموزشي بیماران و خانواده‌های آنان در طول 3 ماه در انجمن مولتیپل اسکلروزیس شهر اصفهان در سال 1390 برگزار شد. سپس پرسش‌نامه ‌هاي دانش، نگرش و عملکرد جمع‌آوری گردید و داده‌ها با استفاده از آزمون‌هاي آماري ناپارامتریک تجزيه و تحليل شدند. يافته‌ها: آزمون Man-Whitney نشان داد؛ قبل از آموزش، دو گروه از نظر آگاهی با هم همسان بودند، ولی بعد از مداخله آموزشی اختلاف معنی‌داری بین آگاهی دو گروه دیده شد (001/0 > P). در ارتباط با نگرش بیماران، قبل از آموزش آزمون Man-Whitney بین دو گروه همسانی نشان داد، ولی بعد از مداخله آموزشی در گروه آزمون 100 درصد بیماران نگرش مثبتی راجع به موضوع پیدا کرده بودند؛ در حالی که تغییری در وضعیت نگرشی گروه شاهد ایجاد نشده بود. در زمینه عملکرد نیز آزمون Man-Whitney تفاوت معنی‌داری بین دو گروه نشان نداد، ولی بعد از آموزش اختلاف معنی‌دار بود (001/0 > P). نتيجه‌گيري: توانمند سازی بیماران مولتیپل اسکلروزیس منجر به ارتقای سطح دانش، نگرش و عملکرد آنان می‌گردد. اجرای چنین برنامه‌های اثربخشی که با محوریت خانواده انجام می‌گیرند، توصیه می‌شود.

Title: ارزيابي تلفيق پيش ازن زني و فرايند انعقاد و لخته سازي با منعقد كننده پلي‌آلومينيوم كلرايد و كمك منعقد كننده پلي‌الكتروليت كاتيوني در تصفيه فاضلاب بيمارستاني

http://hsr.mui.ac.ir/index.php/jhsr/article/view/622

مقدمه: فاضلاب بيمارستاني گستره‌اي از تركيبات مختلف اعم از داروها، گندزداها و عوامل بيماري‌زا هستند كه به طور عمده در چاه‌هاي جاذب دفع مي‌گردد يا وارد شبکه فاضلاب شهري مي‌شود و در نهایت منجر به بحران آلودگي منابع آب مي‌گردد. هدف از انجام اين مطالعه ارزيابي تلفيق پيش ازن زني و فرايند انعقاد ولخته سازي با منعقد كننده پلي‌آلومينيوم كلرايد و كمك منعقد كننده پلي‌الكتروليت كاتيوني در كاهش بار آلودگي فاضلاب بيمارستاني مي‌باشد. روش‌ها: در اين مطالعه از سيستم آزمايشگاهي شامل مولد ازن با ظرفيت go3/h 8/19، رآكتور واكنش با حجم يك ليتر و دستگاه جارتست با استفاده از منعقد كننده پلي‌آلومينيوم كلرايد و كمك منعقد كننده پلي‌الكتروليت كاتيوني استفاده گرديد و پارامترهاي 5BOD، COD TSS مطابق با روش‌هاي ارایه شده در كتاب روش‌هاي استاندارد براي آزمايش‌هاي آب و فاضلاب بررسي شدند. یافته‌ها: بر اساس نتايج اين تحقيق پلي‌آلومينيوم كلرايد با دوز 200 ميلي‌گرم در ليتر همراه با پلي‌الكتروليت كاتيوني با دوز 1 ميلي‌گرم در ليتر باعث راندمان حذف 5BOD، COD TSS به ترتيب معادل 0/50، 0/81 و 0/84 درصد گرديد. پيش ازن زني با دوز  go3/h8/19 به مدت 15 دقيقه راندمان حذف 5BOD،  COD TSSرا در فرايند انعقاد و لخته سازي به ترتيب 0/54، 0/87 و 0/89 درصد حاصل نمود. نتیجه‌گیری: پيش ازن زني كارايي فرايند انعقاد و لخته سازي با منعقد كننده پلي‌آلومينيوم كلرايد و كمك منعقدكننده پلي‌الكتروليت كاتيوني را در حذف پارامترهاي مورد بررسي به ميزان اندكي افزايش داد و همچنين ازن زني ميزان مصرف ماده منعقد كننده پلي‌آلومينيوم كلرايد را كاهش داد.

Title: بررسي سميت نانو ذرات اکسید روی تماس يافته با رنگ آبي 29 با استفاده از دافنيامگنا

http://hsr.mui.ac.ir/index.php/jhsr/article/view/623

مقدمه: مسأله آلودگي محيط زيست به نانو ذرات که به تازگی در ايران وارد چرخه فرايندي صنايع گرديده است، از اهميت چشم‌گيري برخوردار می‌باشد. يكي از اين تركيبات پركاربرد نانو ذرات اکسید روی هستند كه در اين تحقيق، 50LC 24 و 48 ساعته نانو ذرات اکسید روی تماس يافته با رنگ آبي 29 از طريق زيست ‌آزموني اندازه‌گيري شد. روش‌ها: در ابتدا دافينا جهت انجام زيست آزموني در داخل يک ظرف شيشه‌اي كشت داده ‌شد. سپس با انجام آزمايش‌های دامنه‌يابي، گستره غلظت‌هاي مورد نظر تعیین شد و هر غلظت در زمان‌هاي 24 و 48 ساعته براي نانو ذرات اکسید روی به تنهايي و نانو ذرات اکسید روی تماس يافته با رنگ آبي 29، مورد آزمايش قرارگرفت. روش انجام آزمايش‌های زيست آزموني بر اساس روش‌های استاندارد انجام گرفت. جهت تعیین میزان مرگ دافنیا در این مطالعه از آنالیز پروبیت نرم‌افزار 16SPSS استفاده گردید و مقادیر 50LC در زمان‌های مختلف مشخص شد. سپس داده‌های حاصل از آنالیز پروبیت جهت ترسیم نمودارهای مربوط توسط نرم‌افزار Excel مورد استفاده قرار گرفت. یافته‌ها: یافته‌ها نشان داد كه مقدار 50LC 24 ساعته نانو ذرات اکسید روی بدون تماس و تماس یافته با رنگ آبي 29 به ترتيب 6/2 و 8/1 ميلي‌گرم در ليتر و مقادیر 48 ساعته به ترتيب 9/1 و 2/1 ميلي‌گرم در ليتر مي‌باشد. مقدار NOEC 24 ساعته نانو ذرات اکسید روی بدون تماس و تماس یافته با رنگ آبي 29 به ترتيب 9/0 و 2/0 ميلي‌گرم در ليتر و مقادیر 48 ساعته به ترتيب 36/0 و 01/0 ميلي‌گرم در ليتر مي‌باشد. نتیجه‌گیری: با افزايش زمان تماس، مقدار 50LC کاهش یافت؛ به عبارت ديگر سميت نانو ذرات اکسید روی بر ‌روي دافنيا افزایش یافت؛ در نتيجه سميت نانو ذرات تماس يافته با رنگ آبي 29 وابسته به زمان تماس با دافنيامگنا مي‌باشد.

Title: بررسي آگاهي مديران واحدهاي تابعه دانشگاه علوم پزشكي همدان در خصوص كاربردهاي سيستم اطلاعات بيمارستاني

http://hsr.mui.ac.ir/index.php/jhsr/article/view/263

مقدمه: مديران و برنامه‌ريزان بهداشتي درماني كشور وظيفه سياست‌گذاري و برنامه‌ريزي مجموعه ساختاري و نظام بهداشت ملي را به عهده دارند. بي شك براي تسريع و تحقق چنين آرماني، برخورداري ايشان از اطلاعات دقيق و مطمئن كه به راحتي قابل دسترس باشد، ضروري است. از این رو بديهي است كه آگاهي آن‌ها از كاربردهاي مختلف سيستم‌هاي اطلاعات بيمارستاني از اهميت فوق‌العاده‌اي برخوردار باشد. اين مطالعه با هدف تعيين ميزان آگاهي مديران دانشگاه علوم پزشكي همدان در خصوص كاربردهاي سيستم اطلاعات بيمارستان در سال 1390 طراحي و اجرا شد. روش‌‌ها: این مطالعه توصیفی تحلیلی به روش مقطعی در مورد 40 نفر از مدیران مراکز درمانی انجام شد. برای گردآوري داده‌ها از پرسش‌نامه پيش ساخته‌ای شامل 31 سؤال که پایایی و روایی آن تأیید شده بود، استفاده شد. برای توصیف اطلاعات از آمار توصيفي و شاخص‌های مرکزی و پراکندگی و برای تحلیل داده‌ها از آزمون Kruskal-Wallis استفاده شد. یافته‌ها: 5/77 درصد مدیران دانشگاه علوم پزشکی همدان دارای آگاهی متوسط و 5/12 درصد دارای آگاهی خوب در خصوص کاربردهای مدیریتی HIS بودند. همچنین 7/75 درصد از مدیران دوره‌های آموزشی مرتبط را گذرانده بودند. مسؤولین داروخانه بیشترین و مسؤولین رادیولوژی دارای کمترین آگاهی بودند. نتيجه‌گیری: نتایج این مطالعه نشان داد که جهت استفاده بهتر و کاربردی از سیستم اطلاعات بیمارستانی در تمام قسمت‌ها، برگزاری کلاس‌ها و دوره‌های آموزشی علمی ضرورت است.

Title: بررسی عوامل خطرساز دیابت بارداری در زنان باردار مراجعه‌کننده به مراکز بهداشتی درمانی اصفهان

http://hsr.mui.ac.ir/index.php/jhsr/article/view/272

مقدمه: دیابت بارداری یک بیماری شایع ولی بدون علامت و در عین حال پرعارضه می‌باشد. با توجه به اهمیت عوامل خطرساز این بیماری، مطالعات محدود در این زمینه وجود دارد. تفاوت در نتایج آن‌ها، پژوهش حاضر با هدف تعیین عوامل خطرساز دیابت بارداری در زنان باردار مراجعه‌کننده به مراکز بهداشتی درمانی شهر اصفهان سال 1390 انجام شد. روش‌‌ها: در این مطالعه مقطعی پرونده 2014 زن باردار بررسی شد. این افراد ابتدا تحت آزمون غربالگری همگانی یک ساعته GCT (Glucose challenge test) قرار گرفتند و در صورت دارا بودن قند خون mg/dL 140< با آزمون تشخیصی GTT (Glucose tolerance test) و بر اساس ملاک‌های تشخیصی کارپنترو کوستون، موارد دیابت بارداری شناسایی شد. اطلاعات مورد نیاز شامل مشخصات دموگرافیک، عوامل خطرساز دیابت بارداری و نتایج آزمایش‌های قند خون از طریق مستندات مندرج در پرونده، استخراج و به وسیله پرسش‌نامه جمع‌آوری گردید. داده‌ها به دست آمده با نرمافزار SPSS پردازش و با آزمون ANOVA و آزمون Exact fisher و Logistic regression analysis تجزیه و تحلیل شدند. یافته‌ها: از مجموع 2014 خانم مورد بررسی، 77 نفر مبتلا به دیابت بارداری بودند (8/3 درصد). از بین عوامل خطرساز، دیابت بارداری با سن، BMI (Body mass index)، تعداد بارداری، سابقه دیابت فامیلی، مرده‌زایی و زایمان نوزاد ناهنجار رابطه آماری معنی‌دار داشت. آنالیز Logistic regression نشان داد که از بین موارد فوق دیابت بارداری تنها با سن، BMI و سابقه دیابت فامیلی ارتباط آماری معنی‌دار داشت (05/0 > P). نتیجه‌گیری: مهم‌ترین عوامل خطرساز دیابت بارداری به ترتیب شامل سن، چاقی و سابقه دیابت فامیلی می‌باشد.

Title: عنوان: مقایسه کیفیت زندگی زنان و مردان شاغل در شرکت ساپکو

http://hsr.mui.ac.ir/index.php/jhsr/article/view/273

مقدمه: سلامت محیط کار و سلامت روان با ایجاد شاخص‌هاي کیفیت زندگی فراهم می‌شود. توجه به این مسأله در تمام سازمان‌ها ضروري می‌باشد و مانع فرسودگی و راندمان پایین کار خواهد شد. از این رو پژوهش حاضر با هدف مقایسه کیفیت زندگی زنان و مردان شاغل در ساپکو با استفاده از پرسش‌نامه کیفیت زندگی سازمان‌ها جهانی بهداشت (The world health organization quality of life-BREF یا WHOQOL-BREF) در سال1390 انجام گرفت. روش‌‌ها: این مطالعه از نوع توصیفی، تحلیلی و مقطعی است. جامعه آماری زنان و مردان شاغل در شرکت ساپکو بود. نمونه‌گیری تصادفی بر اساس ساختار و ترکیب فیزیکی حضور شاغلین زن و مرد انجام گرفت. برای تجزیه و تحلیل داده‌ها از از آزمون 2c، ANOVA، t-test و نرم‌افزار 5/11SPSS استفاده شد. یافته‌ها: رابطه معنی‌داری بین جنسیت با وضعیت تأهل، داشتن فرزند، درامد و جایگاه شغلی دیده شد ولی رابطه معنی‌داری بین جنسیت با سن، تحصیلات، وضعیت مسکن و سابقه کار دیده نشد. همچنین رابطه معنی‌داری بین کیفیت زندگی با جنسیت، جایگاه شغلی دیده شد ولی رابطه معنی‌داری بین کیفیت زندگی با سن، تحصیلات، وضعیت تأهل، داشتن فرزند، درامد، وضعیت مسکن و سابقه کار دیده نشد. بحث و نتیجه‌گیری: نتایج پژوهش حاضر بیانگر بالاتر بودن کیفیت زندگی زنان از مردان است. برای ارتقای کیفیت زندگی این قشر از جامعه برنامه‌ریزی‌های آموزشی و اجرایی لازم به نظر می‌رسد.

Title: بررسی تأثیر مداخله آموزشی مبتنی بر توانمند سازی سالمندان بر اساس الگوی خانواده محور

http://hsr.mui.ac.ir/index.php/jhsr/article/view/366

مقدمه: سالمندي دوران حساسي از زندگي بشر است و توجه به مسایل و نيازهاي اين مرحله يك ضرورت اجتماعي می‌باشد. در نظر داشتن نيازهاي خاص اين دوران و توجه به توانمند سازي، عزت نفس و خودکارامدی در سالمندان امر بسيار مهمي است كه به طور عمده مورد غفلت قرار مي‌گيرد. اين مطالعه با هدف تعیین تأثير مداخله آموزشی خانواده محور بر توانمند سازی سالمندان با تأکید بر خودکارامدی، عزت نفس، تهدید درک شده و کيفيت زندگي سالمندان شهر اصفهان انجام گرفت.   روش‌ها: در اين مطالعه نیمه تجربي، 64 نفر سالمند داراي توانايي انجام کارهاي معمول و روزانه بدون وابستگي به ديگران و فاقد بيماري‌هاي سيستميک و محدود کننده، به روش نمونه‌گیری در دسترس انتخاب و به دو گروه آزمون و شاهد تقسيم شدند. برای گروه آزمون، الگوي توانمند سازي خانواده محور در 10 جلسه 45 دقیقه‌ای آموزش و اجرا گرديد. سطح تهدید درک شده، عزت نفس، خودکارامدی و کيفيت زندگي هر دو گروه، قبل از مداخله و در پايان مداخله به وسيله پرسش‌نامه ارزيابي شد و در نهایت از آزمون‌هاي 2c، Paired-t و Independent-t جهت تجزيه و تحليل آماري استفاده گردید.   يافته‌ها: آزمون آماری Paired-t بیانگر تأثیر به کارگیری الگوی توانمند سازی خانواده محور در افزایش معنی‌دار سطح کیفیت زندگی گروه آزمون در مقایسه با گروه شاهد بود (01/0 > P). آزمون آماری Independent-t اختلاف معنی‌داری را در دو گروه آزمون و شاهد بعد از اجرای برنامه در نمره عزت نفس (01/0 > P) نشان داد، اما خودکارامدی سالمندان بر خلاف افزایش نسبت به قبل از مداخله، معنی‌دار نبود (07/0P = )؛ در حالی که قبل از مداخله این اختلاف معنی‌دار نبود . آزمون Independent-t در زمینه خودکارامدی تفاوت معنی‌داری بین دو گروه قبل از آموزش نشان نداد و بر خلاف افزایش نمره خودکارامدی بعد از مداخله این اختلاف معنی‌دار نبود (07/0P = ).   نتيجه‌گيري: الگوي توانمند سازي خانواده محور در سالمندان منجر به ارتقای سطح خودکارامدی، عزت نفس، تهدید درک شده و در نهایت کيفيت زندگي سالمندان گردید. اجرای برنامه‌هایی با محوریت خانواده، در این گروه سنی توصیه می‌گردد.

Title: بررسی وضعیت نگرش مذهبی دانشجویان دانشکده بهداشت دانشگاه علوم پزشکی اصفهان

http://hsr.mui.ac.ir/index.php/jhsr/article/view/363

مقدمه: شواهد پژوهشی نشان می‌دهد که نگرش مذهبی در تمام جنبه‌های روان‌شناختی زندگی بشر تأثیرات بسزایی دارد. این پژوهش با هدف، بررسی وضعیت نگرش مذهبی دانشجویان جدید الورود دانشکده بهداشت دانشگاه علوم پزشکی اصفهان انجام گرفت. روش‌ها: مطالعه توصیفی- مقطعی حاضر در مهر ماه 1389 بر روی 200 نفر از دانشجویان جدید الورود دانشکده بهداشت اجرا گردید. داده‌ها با استفاده از پرسش‌نامه نگرش مذهبی دکتر خدایاری‌فرد جمع‌آوری شد و با استفاده از آزمون آماری t و ANOVA تجزیه و تحلیل گردید. یافته‌ها: میانگین و انحراف معیار نمره دینداری کل برای دانشجویان مورد بررسی 337/52 ± 51/486 بود. میانگین و انحراف معیار شناخت دینی 14/5 ± 38/59 و التزام به وظایف دینی 232/29 ± 96/231 به دست آمد. نتایج نشان داد که نگرش مذهبی دانشجویان در تمام ابعاد دینی بسیار قوی و نگرش مذهبی دختران دانشجو به طور معنی‌داری بیش از پسران بود (05/0 > P). همچنین بین متغیرهای سن و رشته تحصیلی با نگرش مذهبی ارتباط معنی‌دار آماری وجود داشت (05/0 > P). نتیجه‌گیری: اجرای برنامه‌های کاربردی توسط دانشگاه به منظور ارتقای بیشتر نگرش مذهبی دانشجویان به خصوص در مؤلفه التزام به وظایف دینی توصیه می‌گردد.

Title: بررسی میزان تأثیر آموزش مبتني بر الگوي فرانظری بر فعالیت جسمانی در دانشجویان

http://hsr.mui.ac.ir/index.php/jhsr/article/view/278

مقدمه: مداخلات آموزشی جهت ارتقای فعالیت جسمانی دانشجویان از اولویت‌های بهداشتی است. یکی از رویکردهای نظری برای ارتقا و نگهداشت فعالیت جسمانی، الگوی فرانظری (Trans-theoretical model) است. این پژوهش با هدف تعیین میزان تأثیر الگوی آموزشی فرانظری بر فعالیت جسمانی دانشجویان در سال 1390 اجرا شد. روش‌‌ها: این پژوهش یک مطالعه نیمه تجربی است که با مشارکت 200 دانشجو (در دو گروه مداخله و شاهد) انجام شد. ابزار جمع‌آوري داده‌ها پرسش‌نامه‌اي چهار قسمتي مشتمل بر اطلاعات فردي‏، پرسش‌نامه مراحل تغییر فعالیت جسمانی (SECQ)، مقياس سنجش خودکارامدی و مقياس سنجش موازنه تصمیم‌گیری بود. مداخله آموزشی مبتنی بر الگوی فرانظری با روش‌های بحث گروهی و سخنرانی اجرا شد. بسته آموزشی شامل پمفلت، پوستر و فیلم بود. 8 هفته پس از اجراي آخرين جلسه آموزشي، داده‌های پس آزمون جمع‌آوری شد. داده‌ها با استفاده از نرم‌افزار 19SPSS و با شاخص‌های مرکزی و آزمون‌های McNemar و Paired t با سطح اطمینان 95 درصد تجزیه وتحلیل شد. یافته‌ها: پیش از مداخله هیچ‌گونه اختلاف معنی‌داری بین شغل، وضعیت تأهل، میانگین سن افراد و مراحل تغییر فعالیت جسمانی در دو گروه آزمون و شاهد وجود نداشت (05/0 < P). در گروه آزمون مراحل فعالیت جسمانی به سمت مراحل فعال پیش رفت (001/0 > P)؛ اما در گروه شاهد مراحل فعالیت جسمانی تغییر معنی‌داری نداشت. نمره موازنه تصمیم‌گیری، خودکارامدی و میزان فعالیت جسمانی در گروه آزمون بر اثر آموزش افزایش معنی‌داری داشت (001/0 > P). نتیجه‌گیری: نتایج به دست آمده نشان داد که اجرای مداخلات آموزشی مبتنی بر الگوی فرانظری یکی از راهکارهای مؤثر در جهت ارتقای فعالیت جسمانی در دانشجویان است.

Title: بررسی دانش، بینش و عملکرد پزشکان عمومی شهر رفسنجان نسبت به سیستم پژوهش سلامت و ارایه راهکارهایی به منظور افزایش سطح دانش، بینش و عملکرد آن‌ها

http://hsr.mui.ac.ir/index.php/jhsr/article/view/733

مقدمه: نظام پژوهش سلامت، نظام برنامه‌ریزی، هماهنگی، پایش و مدیریت منابع و فعالیت‌های پژوهش سلامت به منظور دستیابی به سلامت توأم با عدالت است. پزشکان عمومی به عنوان اعضای اصلی نظام سلامت، نقش به سزایی در دستیابی به آرمان بهداشت و درمان دارند. مطالعه حاضر به منظور بررسی وضعیت دانش، بینش و عملکرد پزشکان عمومی در مورد پژوهش در حیطه سلامت طراحی گردید. روش‌ها: این مطالعه توصیفی بر روی کلیه پزشکان عمومی شهر رفسنجان (76 نفر) انجام شد. اطلاعات به کمک پرسش‌نامه پژوهشگرساخته و به روش سرشماری جمع‌آوری گردید. روایی صوری پرسش‌نامه طبق نظر دو متخصص اپیدمیولوژی قابل قبول بود و پایایی آن به وسیله تکرار آن در یک نمونه کوچک و از طریق محاسبه Cronbach's alpha به دست آمد. داده‌ها با استفاده از آزمون‌های پارامتریک Correlation،ANOVA و t و غیرپارامتریک 2c مورد تجزیه و تحلیل قرار گرفت. یافته‌ها: هفتاد و دو نفر (7/94 درصد) به سؤالات پاسخ دادند که از این بین 39 نفر (1/54 درصد) مرد و 33 نفر (8/45 درصد) زن بودند. در قسمت دانش، 18 نفر (25 درصد) از دانش ضعیف، 39 نفر (2/54 درصد) از دانش متوسط و 15 نفر (8/20 درصد) از دانش خوب برخوردار بودند. در قسمت بینش، 5 نفر (7 درصد) از افراد تحت بررسی از بینش ضعیف، 42 نفر (3/58 درصد) از بینش متوسط و 25 نفر (7/34 درصد) از بینش خوب نسبت به نظام تحقیقات سلامت برخوردار بودند. در مجموع عملکرد افراد شرکت‌ کننده نیز ضعیف ارزیابی شد. نتیجه‌گیری: یافته‌های این پژوهش نشان داد که آگاهی و بینش پزشکان عمومی شهر رفسنجان در مورد سیستم تحقیقات سلامت در حد متوسط و عملکرد آن‌ها ضعیف بوده است. توصیه می‌شود برنامه‌ریزی‌های لازم در جهت ارتقای سطح دانش و بینش پزشکان عمومی در مورد سیستم تحقیقات سلامت از طریق برگزاری کلاس‌ها و کارگاه‌های آموزشی انجام گیرد. همچنین، در نظر گرفتن تسهیلات لازم و بودجه کافی برای پزشکانی که به امر تحقیق در زمینه سلامت می‌پردازند نیز ضروری است.

Title: خودکارامدی: مفهومی عملکردی و تأثیرگذار در کنترل دیابت نوع 2

http://hsr.mui.ac.ir/index.php/jhsr/article/view/345

مقدمه: خودکارامدی به عنوان یک متغیر روانی- اجتماعی در حوزه دیابت، از مواردی است که می‌تواند در انواع پیامدهای این بیماری به صورت مطلوب یا نامطلوب تأثیرگذار باشد. اين پژوهش با هدف تعيين ميزان خودکارامدی و عوامل مؤثر بر آن در بيماران مبتلا به ديابت نوع 2 انجام گرفت. روش‌ها: مطالعه توصیفی- تحلیلی حاضر در سال 1390 به مدت 4 ماه انجام شد. جامعه مورد مطالعه، بيماران مبتلا به ديابت نوع 2 مراجعه كننده به يكي از مراكز ديابت شهر اصفهان بودند. حجم نمونه 140 نفر و به روش نمونه‌گيري مستمر (متوالی) انتخاب شد. ميزان خودکارامدی بيماران با استفاده از پرسش‌نامه دو قسمتی شامل اطلاعات دموگرافیک و مرتبط با بیماری (12 گویه) و ابزار استاندارد خودکارامدی (8 گویه) بر اساس مقیاس ده درجه‌ای لیکرت اندازه‌گیری گردید. داده‌های جمع‌آوری شده با استفاده از نرم‌افزار 11.5SPSS مورد تجزیه و تحلیل قرار گرفت. یافته‌ها: میانگین سنی بیماران 82/7 ± 23/53 سال (75-37 سال) و طول مدت بیماری شرکت کنندگان در این مطالعه 63/5 ± 19/7 سال (20-1 سال) گزارش گردید. میانگین و انحراف معیار خودکارامدی 27/9 ± 29/36 و میزان امتیاز خودکارامدی بین 19 و 56 متغیر بود. در مجموع 6/58 درصد بیماران مبتلا به دیابت دارای خودکارامدی پایین بودند. يافته‌هاي مطالعه نشان داد كه وضعیت تأهل (01/0P < )، سطح تحصیلات (04/0 = P)، وضعیت سلامتی عمومی فرد از دیدگاه خود (04/0 = P)، طول مدت ابتلا به دیابت (02/0 = P) و HbA1C (02/0 = P) با خودکارامدی ارتباط معني‌داري داشتند؛ به طوری که افراد متأهل، دارای تحصیلات بیشتر، وضع سلامتی مطلوب و مدت زمان بیشتر ابتلا به دیابت دارای خودکارامدی بالاتری بودند و با افزایش خودکارامدی میزان HbA1C کاهش یافت. نتیجه‌گیری: با توجه به این مورد که خودکارامدی به عنوان یکی از عوامل مؤثر و کلیدی در کنترل دیابت به شمار می‌رود و این که درصد چشمگیری از بیماران مورد مطالعه از امتیاز خودکارامدی پایینی برخوردار بودند، ضرورت انجام مداخلات آموزشی با بهره‌گیری از مدل‌های آموزش بهداشت و ارتقای سلامت با تمرکز بر سازه خودکارامدی اجتناب ‌ناپذیر می‌نماید.
